# Effects of simulated precipitation gradients on nutrient resorption in the desert steppe of northern China

**DOI:** 10.3389/fpls.2023.1211182

**Published:** 2023-08-30

**Authors:** Liu Bai, Jing Wang, Zhongwu Wang, Zhiguo Li, Haiyan Ren, Haiming Wang, Guogang Zhang, Guodong Han

**Affiliations:** ^1^ Key Laboratory of Grassland Resources of the Ministry of Education, College of Grassland, Resources and Environment, Inner Mongolia Agricultural University, Hohhot, China; ^2^ Key Laboratory of Forage Cultivation, Processing and Higher Efficient Utilization of the Ministry of Agriculture and Rural Affairs, College of Grassland, Resources and Environment, Inner Mongolia Agricultural University, Hohhot, China; ^3^ Center for Comprehensive Test and Demonstration, Inner Mongolia Academy of Agricultural and Animal Husbandry Sciences, Hohhot, China; ^4^ College of Life Sciences, Tianjin Normal University, Tianjin, China

**Keywords:** simulated precipitation, nutrient concentration, nutrient resorption, soil water availability, desert steppe

## Abstract

**Background:**

Changes in rainfall induced by climate change will likely influence the utilization of water resources and affect the nutrient cycle in plants in the water-limited desert steppe. In order to understand the response of nitrogen and phosphorus resorption characteristics of plant leaves to precipitation changes, this study compared the nitrogen (N) resorption efficiency, phosphorus (P) resorption efficiency and influencing factors of plants in a desert steppe through water treatment experiments.

**Methods:**

A 4-year field experiment was performed to examine the response and influencing factors of nitrogen (N) and phosphorus resorption efficiency of five dominant plants in *Stipa breviflora* desert steppe to simulated precipitation change in Inner Mongolia, with four simulated precipitation gradients including reducing water by 50%, natural precipitation, increasing water by 50%, increasing water by 100%.

**Results:**

Compared with natural precipitation, increasing water by 100% significantly increased soil moisture, and significantly increased the aboveground biomass of *S. breviflora*, *C. songorica*, *A. frigida*, decreased the N concentrations in green leaves of S. breviflora, *Cleistogenes songorica*, *Artemisia frigida*, *Kochia prostrata*, decreased the N concentrations in senesced leaves of C. songorica, decreased the P concentrations in green leaves of *K. prostrata* and *Convolvulus ammannii*, decreased the NRE of *S. breviflora*. NRE was significantly negatively correlated with N concentration in senesced leaves, and PRE was significantly negatively correlated with P concentration in senesced leaves.

**Conclusions:**

Increasing water indirectly reduces NRE by reducing plant leaf green leaves nitrogen concentration, and decreasing water indirectly reduces PRE by reducing soil moisture.

## 1 Introduction

Nutrient resorption efficiency refers to the physiological process of perennial plants transporting nutrients from aging tissues to new tissues. This process is conducive to reducing nutrient loss during plant litterfall, prolonging the storage time of nutrients in plants, promoting plant regeneration, and providing nutrients for plant regeneration (Liang et al., 2022). Plants can effectively cycle nutrients in plants through nutrient resorption, thereby reducing nutrient loss, which is conducive to reducing the dependence of plants on soil nutrients, and can quickly and effectively adapt to the growth environment with insufficient nutrient supply, that is, the stronger the nutrient resorption ability, the lower the dependence of plants on soil nutrients, and the more able to adapt to nutrient-poor habitats (May and Killingbeck, 1992). Nitrogen (N) and Phosphorus (P) are key elements for plant growth and development necessary for the synthesis of biological macromolecules. In the process of plant senescence, the resorption of these nutrients is very important for the subsequent growth and development of plants (Zhang et al., 2004; Han et al., 2005).

Nutrient resorption efficiency is affected by precipitation, soil nutrient status and nutrient content of plant leaves (Zhao et al., 2016). Precipitation can affect the availability of soil nutrients, thereby changing the efficiency of plant nutrient resorption efficiency. There is an established significant negative correlation between nutrient resorption efficiency and soil nutrients (Yuan and Chen, 2009). Perhaps unsurprisingly, nitrogen resorption efficiency (NRE) and phosphorus resorption efficiency (PRE) decrease with the increase of soil inorganic nitrogen and available phosphorus (Kang et al., 2019). This has consequences for leaf nutrient content down the line. Globally, there is a significant negative correlation between plant nutrient resorption efficiency and mature green leaf nutrient content (Kobe et al., 2005). The higher the N and P concentration of mature leaves, the higher the NRE and PRE (Zhou et al., 2016). By analyzing the characteristics of plant nutrient resorption efficiency in desert steppe, some studies have shown that there is a significant negative correlation between nutrient content and nutrient resorption efficiency of mature leaves (Zhang et al., 2015).

Global climate change is expected to increase precipitation at the middle latitudes (Intergovernmental Panel on Climate Change (IPCC), 2013). Since the mid-1980s, precipitation in central and northwest China has shown an increasing trend (Zhao et al., 2020). Since 2018, in Inner Mongolia, China, precipitation has increased by 40 mm every decade (Jiao et al., 2022). Change in precipitation will affect the ecosystem structure and function of drylands. Water is the main limiting factor affecting grassland ecosystems (Bai et al., 2008; Yuan and Chen, 2009; Beier et al., 2012). Water availability affects nutrient availability by changing physiological and metabolic responses over a relatively short time scale (for example, 5 years or less) (Smith et al., 2009). On the one hand, the availability of water directly affects plant photosynthetic characteristics (such as stomatal conductance), so as to stimulate plant photosynthesis and changing plant nutrient use efficiency (Ren et al., 2011; Dijkstra et al., 2016). On the other hand, the influence of water on soil mineralization and litter decomposition may also directly affect the availability of soil nutrients, which consequently causes indirect change in plant nutrients (Wang et al., 2018).

These studies have also begun to connect precipitation change to soil moisture and soil available N. The more precipitation, the higher soil available N (Drury et al., 2003; Wu et al., 2021). Some studies have suggested that the increase of precipitation helps to stimulate soil microbial activity, which in turn increases the availability of N in soils (Ren et al., 2015; Xu et al., 2016). In some cases, the increase of soil N availability further increased plant N concentration (Lü et al., 2013; Zhan et al., 2013). However, some studies have found that the soil N content decreases with the increase of water content (Barrett et al., 2002; Chen et al., 2017). This is because even if the increase of precipitation improves the soil mineralization ability, the increase of plant aboveground biomass, organic matter and microbial biomass will lead to the increase of soil microbial N fixation (Lü et al., 2015; Huang et al., 2018). Therefore, with the increase of water, the nutrient resorption efficiency of plant leaves may also decrease (Vinton and Burke, 1995; Murphy et al., 2002). The study of nutrient resorption efficiency in different precipitation gradients showed that the nutrient resorption efficiency decreased from arid to humid areas (Reed et al., 2012; Meier and Leuschner, 2014; Zhao et al., 2016). In one such artificially controlled precipitation experiment, the addition of water reduced the NRE of plant leaves (Lü and Han, 2010). This is consistent with analysis at a global scale which has suggested a significant negative correlation between annual average precipitation and plant NRE (Yuan and Chen, 2009). NRE decreased with the increase of precipitation, while PRE increased with the increase of precipitation, the differential effect of precipitation on soil nutrient status and utilization. Still other studies have shown that both NRE and PRE decrease with the increase of precipitation (Kost and Boerner, 1985; Vergutz et al., 2012). The study of NRE and PRE of typical steppe plants in Inner Mongolia under drought environment found that drought environment reduced plant NRE and PRE (Luo et al., 2018). The study of plant nutrient resorption efficiency under different precipitation gradients in the Qiangtang Plateau found that precipitation indirectly affected the NRE of *Stipa purpurea* by changing the availability of soil nutrients. With the increase of precipitation, the availability of soil nutrients increased and NRE decreased (Zhao et al., 2016).

In arid and semi-arid desert steppe, plant growth is usually limited by water supply, and plant nutrient resorption and utilization will affect the adaptability of plant populations to the environment (Yuan and Chen, 2015; Lü et al., 2019). At present, there is a lack of research on the response of nutrient resorption efficiency of dominant plants to water change in desert steppe. What is the effect of increasing or decreasing water on nutrient resorption efficiency of dominant plants in desert steppe? Will water addition further reduce plant nutrient resorption efficiency by improving soil nutrient availability? Based on this, in order to understand the adaptation of desert steppe plants to arid and barren habitats, a simulated precipitation experiment was carried out in the *S. breviflora* desert steppe of northern China. The N concentration of plant leaves, the P concentration of plant leaves NRE, PRE, soil moisture, soil available N and soil available P of five dominant plants were investigated, and the factors affecting NRE and PRE in the *S. breviflora* desert steppe were clarified.

## 2 Materials and methods

### 2.1 Plant and soil materials and sources

**Figure 1:**
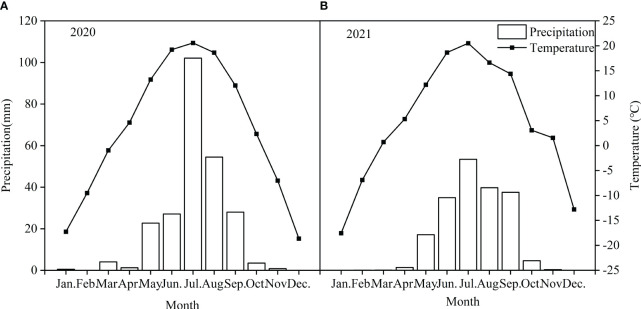
Distribution of precipitation and temperature in the study site in 2020 and 2021.

The study was conducted at a long-term experiment site (41°46′ N, 111°53′ E, 1456 m asl) fenced since 2016, located in a desert steppe in Siziwang Banner in Inner Mongolia, northern China. The site represents a desert steppe which is widely distributed in Inner Mongolia dominated by two perennial grasses (*S. breviflora* and *C. songorica*), two perennial semi-shrubs (*A. frigida* and *K. prostrata*), and one perennial herb (*C. ammanni*). The growing season in this desert steppe runs from early April to late September. At this site, average annual precipitation is 222 mm, 80% of which occurs from May to September (**Figure 1**). Annual precipitation in 2020 was 245 mm and mean temperature was 3.6°C. The total precipitation in 2021 is 189.3 mm, and the average temperature is 4.6°C. The soil is characterized as a Kastanozem (FAO classification) with a sandy loam texture. Mean soil bulk density is 1.3 g·cm^−3^, and pH is approximately 8.0 (top 10 cm). Soil total carbon, total N and total P concentrations for the top 10 cm are 15.1 g·kg^−1^, 1.74g·kg^−1^ and 0.8 g·kg^−1^, respectively.

### 2.2 Experimental design

Beginning in May 2016, 12 plots measuring 4×4 m were established across four different water treatments (Reducing water by 50%, W_-50_; Natural precipitation, W; Increasing water by 50%, W_+50_; Increasing water by 100%, W_+100_) replicated three times. In line with an increasing precipitation trend in western parts of northern China and decreasing precipitation in eastern regions in recent decades (Gao et al., 2015; Sun et al., 2019), addition or reduction of water was applied in the rainy season, which is also the peak season for plant growth. The amount of water applied was determined using methods similar to those used in other related studies (Huang et al., 2016; Li et al., 2018). The water reduction device has a steel frame structure and a transparent V-shaped plastic plate to collect rainwater into a rain collection bucket. Iron sheet fences were built around each treatment community which protrude 10 cm above the ground and extend another 40cm deep to prevent horizontal water loss. The precipitation data was based on average rainfall data collected through the meteorological station at the test site, the corresponding amount for each treatment was sprayed manually at the middle and end of each month Soil moisture was measured from May to October every year. The amount of water applied each month can be found in **Table 1**.

**Table 1:** Simulated precipitation change (mm) in different water treatment in 2020 and 2021.

Year	Treatment	May	June	July	August	September	October	overall amount
2020	W_-50_	11.35	13.55	51.05	27.25	14	1.75	118.95
	W	22.7	27.1	102.1	54.5	28	3.5	237.9
	W_+50_	34.05	40.65	153.15	81.75	42	5.25	356.85
	W_+100_	45.4	54.2	204.2	109	56	7	478.8
2021	W_-50_	8.55	17.45	26.85	19.85	18.75	2.3	93.75
	W	17.1	34.9	53.7	39.7	37.5	4.6	187.5
	W_+50_	25.65	52.35	80.55	59.55	56.25	6.9	281.25
	W_+100_	34.2	69.8	107.4	79.4	75	9.2	375

### 2.3 Sample collection

Soil moisture was measured using a portable soil moisture meter PR2 moisture tube (DEVICES LTD, Delta-T, UK). Three replicates were measured per precipitation treatment cell.

In the fifth and sixth year of the water treatment experiment, soil was sampled with a five-point sampling method. Three soil drills with a diameter of 3 cm and a length of 10 cm were used to collect 0-10 cm deep soil in each precipitation treatment plot, and uniformly mixed into one sample. All samples were divided into two parts. The first part was stored in a refrigerator at 4°C for the determination of soil available nitrogen. The other part was dry to determine soil available P.

Vegetation was investigated with a × 50 cm quadrat in each plot. The number of plant species was recorded and above-ground living plants were harvested. Plants were oven-dried at 65°C for at least 48 h and then weighed.

At the mature stage of green leaves in August 2020 and 2021, five dominant plants were randomly selected in each plot, including *S. breviflora*, *C. songorica*, *A. frigida*, *K. prostrata* and *C. ammannii*. In the experimental year, the five plants accounted for about 70% of the net primary productivity. The size of each selected plant cluster was kept as consistent as possible to reduce age-effects, and labels were inserted near the plant to ensure they were correctly identified in the case of resampling. One of the 3rd or 4th fully expanded leaves at the top of the plant was sampled. We chose whole leaves that showed little sign of pest or pathogen damage. From late September to mid-October, the remaining leaves are monitored on a weekly basis and collected when the leaves become brown and not falling. The collected plant leaves were dried in an oven at 65°C for 48 h and weighed. The dried plant samples were ground by a ball mill and screened with a 40-mesh sieve, which was placed in a self-sealing bag for N and P determination.

### 2.4 Samples measurements

Soil available nitrogen was measured using KCL dipping method. The plant leaves N concentration was determined using an elemental analyzer. The soil available phosphorus and P concentration of plant leaves were determined by molybdenum antimony colorimetry.

### 2.5 Statistical analyses

Resorption efficiency (RE) of plants was calculated as the percentage reduction in nutrient concentration between green and senesced leaves after accounting for a mass loss correction factor (MLCF) (Vergutz et al., 2012):


$$RE\left(\%\right)=\left[1-\left(\frac{Nutrient_{senesced}}{Nutrient_{green}}\right)\times MLCF\right]\times 100$$

where Nutrient_green_ and Nutrient_senesced_ are the N or P concentrations in green and senesced leaves, respectively, and the MLCF (mass loss correction factor) was calculated as the ratio of the dry mass of senesced leaves to the dry mass of green leaves (Van et al., 2003).

The effects of treatments on nutrient concentration and nutrient resorption were analyzed by one-way ANOVAs for each species. All analyses were conducted with SAS version 9.2. In order to accurately distinguish the contribution of precipitation, soil nutrient status and leaf nutrient concentration to leaf resorption efficiency, principal component analysis was used to explore the responses of NRE and PRE to environmental, plant and soil variables. Pearson correlation analysis was carried out using the PROC CORR process to explore any potential correlations between NRE, PRE and soil and plant nutrients. Path analysis of the influencing factors of NRE and PRE using structural equation models. All graphs are drawn using Origin 2021.

## 3 Results

### 3.1 Responses of soil moisture and nutrient availability to water treatment

Reducing water by 50% significantly decreased soil moisture by 21.59% (*P<0.05*, **Table 2**), and increasing water by 50% more significantly increased soil moisture by 22.00% (*P<0.05*, **Table 2**), and a clear increase of 42.58%was also observed in the increasing water by 100% plots (*P<0.05*, **Table 2**). Soil available N (NH_4_^+^plus NO_3_^−^) was significantly greater in plots with reduced water compared with the natural precipitation treatment. Surprisingly, we did not observe the opposite response, and neither the 50% or 100% rainfall addition influenced available soil N.

**Table 2:** Effects of water treatment on soil moisture (%) and soil available N and P concentrations (mg·kg^-1^).

Treatment	Soil moisture	Soil available N	Soil available P
W_-50_	9.30 ± 0.65d	26.39 ± 2.12a	7.53± 0.72a
W	11.86 ± 0.11c	12.48 ± 0.98b	6.37± 0.48ab
W_+50_	14.47 ± 0.27b	13.51 ± 1.35b	6.18± 0.32ab
W_+100_	16.91 ± 1.16a	13.48 ± 0.89b	5.33± 0.52b

Data are means ± SE. Different lowercase letters in each column indicate significant differences at P<0.05. W_-50_, W, W_+50_, W_+100_ refer to reduction water by 50%, natural precipitation, addition water by 50% and addition water by 100%, respectively.

### 3.2 Responses of aboveground biomass to water treatment

Water addition greatly affected the aboveground biomass of plants. Increasing water by 50% significantly increased the aboveground biomass of *S. breviflora* from 29.19 to 67.51 g·m^-2.^ Similarly, *S. breviflora* increased in size from 29.19 to 78.63 g·m^-2^ when given increasing water by 100%. (*P<0.05*, **Table 3**). Similarly, the aboveground biomass of *A. frigida* significantly increased to 55.71 g·m^-2^ in increasing water by 50%, and increased even further to 76.63 g·m^-2^ in the increasing water by 100% experiment (*P<0.05*, **Table 3**). The aboveground biomass of *C. songorica* followed similar patterns, and increased by 66.56% with increasing water by 100%. Similarly, the aboveground biomass of *C. ammannii* increased by 67.53% with only increasing water by 50% (*P<0.05*, **Table 3**). The aboveground biomass of *K. prostrata* were also enhanced in water addition plots. Reducing water by 50% reduced the aboveground biomass of each of the five species.

**Table 3:** Effects of water treatment on aboveground biomass (g·m^-2^).

Species	Treatment	Mean
Sb	W_-50_	9.67 ± 2.87b
	W	29.19 ± 4.87b
	W_+50_	67.51 ± 14.20a
	W_+100_	78.63 ± 11.69a
*Cs*	W_-50_	24.25 ± 1.75b
	W	26.71 ± 1.96b
	W_+50_	26.98 ± 1.71b
	W_+100_	44.49 ± 5.44a
*Ca*	W_-50_	11.51 ± 3.23c
	W	17.31 ± 3.55bc
	W_+50_	29.00 ± 1.95a
	W_+100_	23.49 ± 3.03ab
*Af*	W_-50_	12.56 ± 4.79b
	W	34.76 ± 9.05b
	W_+50_	90.47 ± 18.58a
	W_+100_	111.39 ± 23.28a
*Kp*	W_-50_	10.69 ± 3.06a
	W	11.86 ± 4.35a
	W_+50_	18.80 ± 4.38a
	W_+100_	24.10 ± 7.12a

Data are means ± SE. Different lowercase letters in each column indicate significant differences at P<0.05. Sb, Stipa breviflora; Cs, Cleistogenes songorica; Ca, Convolvulus ammannii; Af, Artemisia frigida; Kp, Kochia prostrata.

### 3.3 Responses of plant N and P concentrations to water treatment

**Figure 2:**
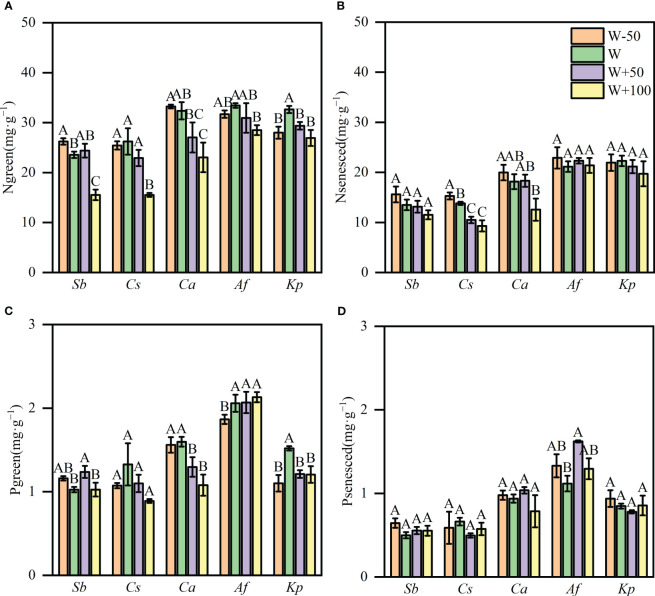
Plant nitrogen (N) and phosphorus (P) concentrations in response to water treatment in a desert steppe. **(A, B)** N concentrations in green and senesced plant leaves. **(C, D)** P concentrations in green and senesced plant leaves. Data are means ± SE. Different lowercase letters in each column indicate significant differences at *P*<0.05.

Water reduction and water addition affected N and P concentrations in both green and senesced leaves. Compared with natural precipitation treatment, reducing water by 50% significantly increased the green leaf N concentration of *S. breviflora* by 11.3%. Compared with the natural precipitation treatment, N concentrations in green leaves across *K. prostrata* reduced from 32.63mg·g^−1^ to 29.93 mg·g^−1^ in the increasing water by 50% plots (*P<0.05*, **Figure 2A**). N concentrations dropped significantly in the 100% addition water plots, and green leaves across *S. breviflora*, *C. songorica*, *C. ammannii*, *A. frigida* and *K. prostrata* reduced their N content by 34.09%, 40.79%, 25.67%, 14.75% and 17.47%, respectively. (*P<0.05*, **Figure 2A**). N concentration in senesced leaves of five plants ranged from 9.30 to 22.88 mg·g^−1^. Compared with the natural precipitation treatment, reducing water by 50% significantly increased the senesced leaves N concentration of *C. songorica* by 10.56%. Increasing water by 50% and 100% significantly reduced the N concentration of senesced leaves of *C. songorica* by 24% and 32.69%, respectively (*P<0.05*, **Figure 2B**). P concentrations were reduced by adding water in *C. ammanii* as well, with a decrease of 0.30 mg·g^−1^ and 0.52 mg·g^−1^ for the increasing water by 50% and 100% treatment, respectively (*P<0.05*, **Figure 2B**). The 50% increase in water access increased the P concentration in green leaves of *S. breviflora*, and reducing water by 50% decreased the P concentration in green leaves of *A. frigida* (*P<0.05*, **Figure 2C**). Compared with natural precipitation treatment, addition water by 50% significantly increased the phosphorus concentration in senesced leaves of *A. frigida* by 45.23% (*P<0.05*, **Figure 2D**).

### 3.4 Responses of plant N and P resorption efficiency to water treatment

**Figure 3:**
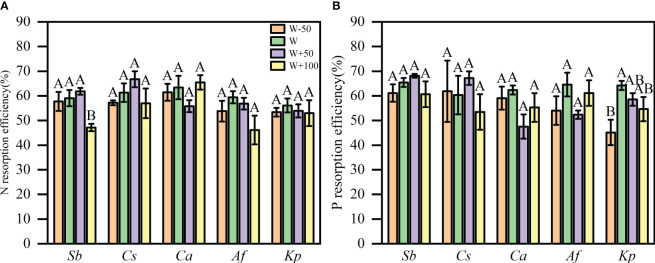
Plant nitrogen resorption efficiency (NRE) in response to water treatment **(A)**. Plant phosphorus resorption efficiency (PRE) in response to water treatment **(B)**. Data are means ± SE. Different uppercase letters between treatments indicate the significant difference at 0.05 level.

Both water reduction and water addition produced a decline in NRE (P<0.05, **Figure 3A**), but the response of NRE to water treatment differed among species. NRE shifted from 59% to 47% for S. breviflora leaves in the increasing water by100% plot (P<0.05, **Figure 3A**). The reduction water treatments tended to reduce the NRE of the five plants. (**Figure 3A**).

Compared with natural precipitation, reduction water by 50% treatment significantly reduced the PRE of K. prostrata by 29.64% (P<0.05, **Figure 3B**. When water access was reduced by 50%, the PRE of the other four plants was lower than that of natural precipitation treatment. Interestingly, the PRE of S. breviflora and C. songorica increased when water access was increased by 50%, but decreased under 100% water addition (**Figure 3B**).

### 3.5 Contribution rate of different factors to NRE and PRE

**Figure 4:**
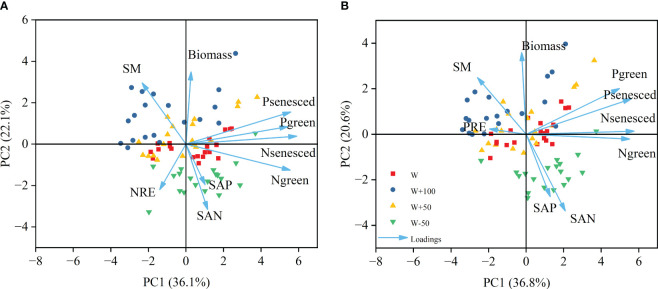
Results of principal component analysis for leaf N concentration, leaf P concentration and resorption efficiency, soil moisture, soil available N and soil available P. The direction of the arrow indicates the maximum increase in the variable, and the length indicates the strength of influence of the selected parameter relative to other variables.

In the principal component analysis of NRE, the first principal component contribution rate was 36.1%, the second principal component contribution rate was 22.1%, and the cumulative contribution rate was 58.2%. (**Figure 4A**).In the principal component analysis of PRE, the contribution rate of the first principal component was 36.8%, the contribution rate of the second principal component was 20.6%, and the cumulative contribution rate was 57.4%. (**Figure 4B**). The nutrient concentration of plant leaves was the first principal component influencing NRE and PRE, soil factors and biomass were the second principal component.

There was a strong positive correlation between green leaf N concentration and senesced leaf N concentration, and similarly between green leaf P concentration and senesced leaf P concentration. Soil moisture was significantly negatively correlated with soil available N, green leaves N concentration and green leaves P concentration. Aboveground biomass was positively correlated with soil moisture, green leaf P concentration and dead leaf P concentration, and negatively correlated with soil available N. Soil available N was significantly positively correlated with soil available P. There was a significant negative correlation between NRE and leaves N concentration and leaves P concentration. There was a significant negative correlation between PRE and leaf P concentration (**Table 4**).

**Table 4:** Correlations between leaf N concentration, leaf P concentration, N and P resorption efficiency, soil moisture and soil available N and soil available P.

Correlation coefficient										
Index	SM	SAN	SAP	Biomass	Ngreen	Nsenesced	Pgreen	Psenesced	NRE	PRE
SM	1									
SAN	-0.27**	1								
SAP	-0.04	0.25**	1							
Biomass	0.42***	-0.25**	-0.17	1						
Ngreen	-0.36***	0.21	0.09	-0.19*	1					
Nsenesced	-0.18	0.17	0.2	-0.11	0.73***	1				
Pgreen	-0.24*	-0.09	-0.09	0.29**	0.72***	0.59***	1			
Psenesced	-0.09	-0.01	0.05	0.29**	0.58***	0.78***	0.71***	1		
NRE	-0.15	0.03	0.09	-0.04	0.2	-0.47***	0.04	-0.35**	1	
PRE	0.52	-0.23*	-0.1	-0.06	0.04	-0.36**	0.15	-0.54***	0.52***	1

The marker *, ** and ***indicates statistically significant at P < 0.05, P < 0.01 and P < 0.001 level.

**Figure 5:**
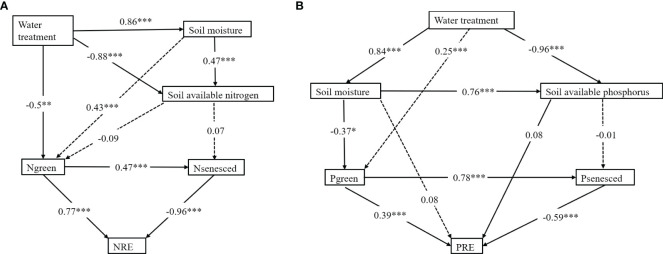
**(A)** The controlling factor analysis of leaf NRE using structural equation modeling. **(B)** The controlling factor analysis of leaf PRE using structural equation modeling. The numbers represent the standard path coefficient; solid lines represent significant effects (P< 0.05); and dotted lines represent non-significant relationships. The marker *, ** and ***indicates statistically significant at P < 0.05, P < 0.01 and P < 0.001 level.

The effects of water treatment, soil nutrients and leaf N and P on leaf NRE and PRE were analyzed using structural equation modeling. The results showed that the direct effects of water treatment on soil moisture and green leaves N concentration were the most significant, respectively, while the direct effects on other factors were small. The direct effect of soil moisture on soil available N was the most significant, implying that changes in soil moisture had little direct effect on other factors. The direct effects of N concentration in green leaves on N concentration and NRE in senesced leaves were the most significant, respectively. The direct effect of N concentration on NRE in senesced leaves was the most significant (**Figure 5A**). The direct effect of water treatment on soil moisture and soil available P was significant. The direct effect of soil moisture on green leaves P concentration was significant. The direct effects of green leaves P concentration on senesced leaves P concentration and PRE were significant (**Figure 5B**). These results indicate that soil moisture is a key factor affecting leaves NRE and PRE. Water treatment indirectly affected NRE and PRE of plant leaves by affecting soil moisture and N and P concentrations in green leaves.

## 4 Discussion

### 4.1 Effect of water on aboveground biomass

Changes in precipitation affect soil moisture, and plants consequently absorb more water to regulate biomass and species composition. In this study, reducing water by 50% decreased aboveground biomass, while increasing water by 50% and 100% increased aboveground biomass. This shows how aboveground biomass of plants is sensitive to change in precipitation, which is consistent with other research results on aboveground biomass in desert grassland in north China (Yang and Liu, 2015). In the arid and semi-arid Stipa breviflora desert steppe, water is an important limiting factor, though a complex one with many interactions. The reduction of water and the poor utilization of soil nutrients affect plant growth, whereas increasing precipitation accelerates the soil nutrient cycle and microbial activity, promotes soil microorganisms, enriches the species composition of plants and increases biomass (Jordan et al., 2020). which shows that increasing water is generally a net positive conducive to increasing aboveground biomass of arid desert grassland plants and plant community productivity.

### 4.2 Effects of water treatment on leaf nutrient status

The N concentration in green leaves of dominant plants in the desert steppe decreased with water addition. Under water addition treatments, the N concentration in green leaves was significantly lower than in the natural precipitation plots. There may be two reasons for this. On the one hand the arid environment increases the osmotic pressure inside the cells of desert steppe plants and enhances the protection of water in the plant by increasing the input of nitrogen in non-photosynthetic organs or tissues inside the leaves, an effect that may be exacerbated as we further diminish the water access of plants (Seligman and Sinclair, 1995) When water increases, water stress gradually reduces and plants no longer need to allocate more nitrogen to non-photosynthetic organs or tissues in leaves, and the nitrogen content in leaves decreases (Huang et al., 2018). On the other hand, the effect of water addition on vegetation growth may be greater than the effect on soil N mineralization (Lü et al., 2013), resulting in the dilution of N concentration in green leaves and decreased leaves N concentration (Yuan and Chen, 2009). We observed that water addition reduces the N concentration of senesced leaves, and water reduction increases the N concentration of senesced leaves, and this observation is likely a product of changes in leaf N status and soil N availability. Generally, it is considered that the higher the soil available N, the higher the plant tissue N concentration (Zhao et al., 2016). However, the climate characteristics of desert steppe may have a significant effect. Annual precipitation is less than annual evaporation, so in order to enhance the protection of water in the plant, the leaves of dominant plants in the desert steppe increase the input of nitrogen to non-photosynthetic organs or tissues inside the leaves and increase the osmotic pressure inside the cells (Seligman and Sinclair, 1995) to adapt to the arid environment, which may explain the high concentration of N in green leaves.

In this study, water addition decreased the P concentration in green leaves and senesced leaves, but water decrease increased the P concentration in green leaves and senesced leaves. The reasons for the different responses of the five plants to water addition may be related to the different needs and utilization modes of the plants themselves. Some studies suggest that water addition reduces the P concentration in plant leaves because water addition promotes the growth of plants and the increase in biomass dilutes the P concentration in leaves (Bai, 2013). Conversely, some other studies have shown that the absorption of P by plants is positively correlated with water conditions, and as such, water addition should increase the P content of plants (Ren et al., 2018; Song et al., 2020). Adjusting the proportion of leaf element concentration at different stages of plant growth is a physiological function of how plants adapt to their habitat, and increase or decrease in P concentration may be the result of continuous mutual regulation between the above two factors. An experimental study on water addition in a typical grassland of Inner Mongolia showed that water addition had no effect on the N concentration of mature leaves but significantly reduced P concentration, while it had a significant effect on the N and P concentration of withered leaves (Lü and Han, 2010: He et al., 2008). This shows that in the typical steppe with good soil water conditions, the effect of water addition on plant P concentration was weakened. This evidence, and our results herein can be taken together to underscore how water conditions likely restrict and stress for the Stipa breviflora desert steppe.

### 4.3 Nutrient resorption efficiency and proficiency in desert steppe species status

Plant nutrient resorption efficiency is an indicator of plant adaptability to the environment. The change of plant nutrient resorption characteristics in desert steppe helps to alleviate the limitation of soil nutrients. The soil nutrient concentration of desert steppe is low, the decomposition of organic matter is slow, and the nutrients produced by litter decomposition contribute less to the nutrient circulation system of desert steppe. Nutrient resorption efficiency is one strategy by which desert plants adapt to these nutrient-poor habitats. The species in this study showed high NRE and PRE. In the process of leaf senescence, the NRE of S. breviflora was 59.08%, PRE was more than 60%, and are also high compared to global grassland data (46.9%) (Yuan and Chen, 2015). We did note that water addition plots reduced NRE. We took this to mean that the high NRE of dominant plants in the desert steppe appears to be based on the reuse of leaf nutrients. The extremely high NRE in the leaves of dominant plants in the desert steppe reflects their adaptability to the extremely arid and nutrient-poor environment, suggesting that adaptation to a low-nutrient environment occurs through a form of internal N cycling (Lu et al., 2018). In order to maintain high N input, plant leaves should have higher NRE especially in extremely arid environments. Therefore, the arid environment in this study area may be the reason for the difference in our results with those of other studies (Li et al., 2010). Reduction water reduced the P resorption efficiency of K. prostrata, and had no significant effect on the other four plants. This may be related to the different needs and utilization modes of the plant itself, or it may be because the PRE of the plants were already very high and not able to further increase due to the constraints of physiological processes.

### 4.4 Placing nutrient resorption efficiency-dependent nutrient cycling in context

The nutrient resorption efficiency of plants is calculated based on the difference between the nutrient content of green leaves and senesced leaves, so the nutrient concentration of leaves has a direct impact on the nutrient resorption efficiency. We observed that NRE was positively correlated with N concentration in green leaves and negatively correlated with N concentration in senesced leaves (Lu et al., 2018). Furthermore, the N concentration in green leaves was proportional to that in senesced leaves. The higher the N concentration in green leaves, the higher the N concentration in senesced leaves (Kobe et al., 2005). NRE was significantly negatively correlated with N concentration in senesced leaves, but not significantly correlated with N concentration in green leaves, indicating that the degree of N resorption in senesced leaves was an intrinsic determinant of NRE, which is unsurprising. The PRE of plant leaves has a negative correlation with soil available P, so increasing water improves soil available P but reduces plant PRE, which is consistent with previous research results (Li et al., 2013). Research results show that senesced leaves’ P concentration is positively correlated with PRE (Zhou et al., 2016). The results of this study are consistent with this finding, indicating that the PRE level of senesced leaves is an internal determinant of PRE. Soil nutrients represents key factors affecting plant NRE and PRE.

The availability of plant soil nutrients in the study area is low, and plant leaves have high nutrient resorption efficiency. In the process of plant leaves withering, more N and P are recycled and stored in roots (Bai, 2013). This strategy can prolong the residence time of nutrient elements in plants. In nutrient poor ecosystems, the longer residence time of N and P is conducive to the maintenance of plant tissue nutrients and can create conditions for plant rejuvenation the following year (Zhao et al., 2016). In the process of senescence, more N and P were resorbed and reused in the leaves of dominant plants in desert grassland, resulting in lower N and P concentrations in senescent leaves (Zhang et al., 2020), hence resorption levels are high.

## 5 Conclusion

Water addition improves soil moisture in the desert steppe, promoting the growth of plants and increasing the biomass of plant communities. This promotes the consumption of soil available nutrients by plants, which in turn draws down finite soil nutrient resources. Increasing water supply reduced plant nutrient resorption efficiency. The resorption of N and P in senesced leaves plays a decisive role in the resorption efficiency of N and P in leaves. Soil nutrient availability is the key factor affecting the leaf N and P resorption efficiency of dominant plants in the desert steppe. Water addition indirectly affects the leaf nutrient resorption efficiency of dominant plants in the desert steppe by affecting soil nutrient availability and leaf nutrient concentrations.

## Data availability statement

The raw data supporting the conclusions of this article will be made available by the authors, without undue reservation.

## Author contributions

LB was responsible for writing the manuscript; ZW was responsible for the design and management of the experiment. JW, ZL, HR, GZ, and GH assisted LB in sampling and determination of physiological and biochemical indexes. HW provided us with grassland. All authors contributed to the article and approved the submitted version.
